# EndOxy: Dynamic Long-Term Evaluation of Endothelialized Gas Exchange Membranes for a Biohybrid Lung

**DOI:** 10.1007/s10439-019-02401-2

**Published:** 2019-11-21

**Authors:** Sarah Klein, Felix Hesselmann, Suzana Djeljadini, Tanja Berger, Anja Lena Thiebes, Thomas Schmitz-Rode, Stefan Jockenhoevel, Christian G Cornelissen

**Affiliations:** 1 Department of Biohybrid & Medical Textiles (BioTex), AME – Institute of Applied Medical Engineering, Helmholtz Institute Aachen, RWTH Aachen University, Forckenbeckstraße 55, 52074 Aachen, Germany; 2 Faculty of Science and Engineering, Aachen-Maastricht Institute for Biobased Materials, Maastricht University, Brightlands Chemelot Campus, 6167 RD Geleen, The Netherlands; 3 Department of Cardiovascular Engineering (CVE), AME – Institute of Applied Medical Engineering, Helmholtz Institute Aachen, RWTH Aachen University, Pauwelsstraße 20, 52074 Aachen, Germany; 4 DWI-Leibniz Institute for Interactive Materials, Forckenbeckstraße 50, 52074 Aachen, Germany; 5 Department of Medical Statistics, RWTH Aachen University Hospital, Pauwelsstraße 19, 52074 Aachen, Germany; 6 Department of Pneumology and Internal Intensive Care Medicine, Medical Clinic V, RWTH Aachen University Hospital, Pauwelsstraße 30, 52074 Aachen, Germany

**Keywords:** Artificial lung, Extracorporeal membrane oxygenation, Gas transfer, Tissue engineering, Whole blood

## Abstract

In the concept of a biohybrid lung, endothelial cells seeded on gas exchange membranes form a non-thrombogenic an anti-inflammatory surface to overcome the lacking hemocompatibility of today’s oxygenators during extracorporeal membrane oxygenation. To evaluate this concept, the long-term stability and gas exchange performance of endothelialized RGD-conjugated polydimethylsiloxane (RGD-PDMS) membranes was evaluated. Human umbilical vein endothelial cells (ECs) were cultured on RGD-PDMS in a model system under physiological wall shear stress (WSS) of 0.5 Pa for up to 33 days. Gas exchange performance was tested with three biological replicates under elevated WSS of 2.5 Pa using porcine blood adjusted to venous values following ISO 7199 and blood gas analysis. EC morphology was assessed by immunocytochemistry (*n* = 3). RGD-PDMS promoted endothelialization and stability of endothelialized membranes was shown for at least 33 days and for a maximal WSS of 2.5 Pa. Short-term exposure to porcine blood did not affect EC integrity. The gas transfer tests provided evidence for the oxygenation and decarboxylation of the blood across endothelialized membranes with a decrease of transfer rates over time that needs to be addressed in further studies with larger sample sizes. Our results demonstrate the general suitability of RGD-PDMS for biohybrid lung applications, which might enable long-term support of patients with chronic lung failure in the future.

## Introduction

With 3 million deaths in 2016, chronic obstructive pulmonary disease (COPD) is the third most frequent cause of mortality worldwide.^35^ COPD is a progressive and incurable lung disease, so treatment is mostly limited to symptomatic relief and improving the quality of life. Exacerbations in patients with severe COPD are often associated with acute respiratory failure and carbon dioxide retention.^8^ When mechanical ventilation is unable to improve gas exchange, extracorporeal membrane oxygenation (ECMO) is an alternative treatment.^28^

During ECMO, the patient’s blood is passed over gas exchange membranes in an oxygenator to remove excess carbon dioxide and to oxygenate the blood.^10^ It is a highly invasive treatment that requires continuous intensive care. Moreover, commercially available oxygenators can only be used temporarily with most studies indicating an average application of 10 to 20 days.^30^ Regular device exchange is necessitated by inflammatory reactions, thrombosis, bleeding, and hemolysis.^28^ These complications reflect the poor hemocompatibility of the gas exchange membrane in direct contact with the blood. Although use of ECMO is increasing worldwide^26^ and various efforts have been made to improve hemocompatibility,^21^ no currently available oxygenator achieves comprehensive long-term support of gas exchange function for months or even years.

Seeding endothelial cells on gas exchange membranes as a biohybrid approach may overcome current limitations by forming a non-thrombogenic and anti-inflammatory surface.^14^ However, cell coating places additional design requirements on the ‘biohybrid lung’. In addition to sufficient gas exchange, an integral endothelium with a durable monolayer of functional endothelial cells must be formed and maintained under blood-flow conditions over a long period. Thus far, the endothelialization of heparin/albumin-coated poly(4-methyl-1-pentene) (PMP) membranes has been achieved for up to 4 weeks of static cultivation^9^,^37^ as well as the promising first results of dynamic cultivation for 2 weeks (review article).^20^ Furthermore, short-term dynamic cultivation (hours to days) has also been demonstrated for various surface modifications and gas exchange membranes such as TiO_2_-coated PMP,^22^ fluorocarbon membranes^5^,^15^ and polypropylene^32^ or silicone membranes^27^ displaying immobilized peptides or proteins. However, the stability of endothelialized gas exchange membranes for extended periods under well-defined flow conditions, as well as their gas exchange performance under standardized conditions in blood, has not been studied in detail.

We therefore investigated the long-term stability of an endothelialized membrane in a microfluidic model system under well-defined physiological wall shear stress (WSS) conditions for up to 33 days and with three independent biological replicates. Although the intended use of a biohybrid lung ranges from several months to even years, the culture period of 33 days was chosen to exceed the 4-week approval of today’s clinical oxygenators.

To investigate the influence of the cell layer on gas exchange performance in dynamic culture, the gas exchange performance of the endothelialized membranes was subsequently tested in whole blood following ISO 7199 for blood–gas exchangers.^6^ These tests were performed with significantly elevated WSS values to determine the stability of the cell layer under more demanding flow conditions.

## Materials and Methods

### Cell Isolation and Culture

Human umbilical vein endothelial cells (HUVECs) were derived from umbilical cords according to established protocols.^15^ Umbilical cords were kindly provided by the Department of Gynecology and Obstetrics (RWTH Aachen University Hospital) following approval by the ethics committee at the medical faculty of the RWTH Aachen University (EK 019) and informed consent provided by the patients. Briefly, the vein was washed with phosphate-buffered saline (PBS, Thermo Fisher Scientific) and HUVECs were isolated by enzymatic dissociation in 1 mg mL^−1^ collagenase (Sigma-Aldrich). These endothelial cells (ECs) were cultured in flasks coated with 2% gelatin and incubated with EC growth medium 2 (EGM-2, PromoCell) in a humidified incubator at 37 °C and 5% CO_2_. Cell passages 3–4 sourced from three different donors were used for all experiments.

### Microfluidic Flow Chamber with RGD-Conjugated Films

Thin polydimethylsiloxane (PDMS) films (100 *µ*m, Wacker Chemie) were thoroughly cleaned using soap and absolute ethanol (Merck), rinsed with ultra-pure water (Sartorius) and air dried. The films were mounted on commercially available microfluidic flow chambers (sticky *µ*-slides I^0.1^, Ibidi) with a chamber height of 150 *µ*m. The assembled devices were placed in an oven at 60 °C for 8 h and allowed to cool to room temperature to prevent membrane detachment. To enable cell attachment, RGD peptides were conjugated to the cell growth area of the PDMS films as previously described,^12^ to generate the RGD-PDMS membranes. The microfluidic channels were disinfected twice with 70% ethanol for 10 min, rinsed with sterile PBS and stored at 4 °C.

### Model System

**Figure 1:**
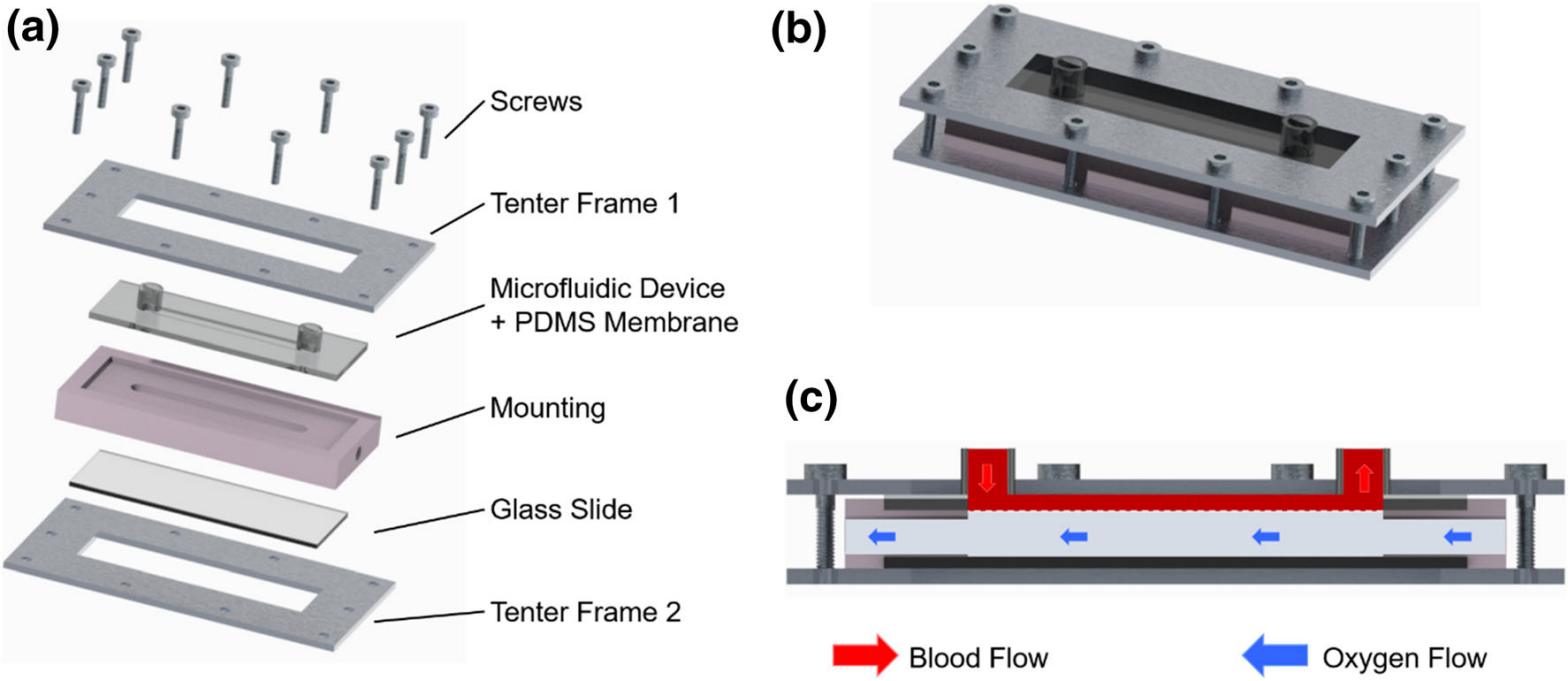
Microfluidic flow chamber placed in a PDMS-cast mounting and secured with a metal tenter frame (a and b). Schematic cross-section of blood interface (c).

For long-term dynamic culture, separate model systems were used for each of the three donors. In each model system, up to four microfluidic flow chambers were connected in series. Each model system comprised a 2-L medium reservoir (Schott), a peristaltic pump (ISMATEC IPC-N 8 with PHARMED ISMAPRENE pump tubes, both Cole-Parmer) as well as disposable tubing (Extension Line Type Heidelberger, B. Braun Melsungen or Fresenius Kabi). The microfluidic flow chambers were placed in PDMS-cast mountings and secured with a metal tenter frame (Figs. 1a and 1b). The mounting also formed the gas pathway for the gas transfer measurements (Fig. 1c).

Before connecting the microfluidic flow chambers, the model system was flushed with ~ 200 mL EGM–2 supplemented with 1% antibiotic-antimycotic solution (ABM, Thermo Fisher Scientific) and equilibrated to incubator conditions (37 °C, 5% CO_2_). During experiments, equilibration to incubator conditions and sufficient gas exchange was guaranteed by the reservoir, which was open to the incubator atmosphere in a sterile manner.

### Static and Dynamic Culture

For the endothelialization of RGD-PDMS films, HUVECs were suspended in EGM-2 with 1% ABM and transferred to the microfluidic channel at a concentration of 8 × 10^6^ cells mL^−1^ (equivalent to 1.2 × 10^5^ cells cm^−2^). Cell-seeded microfluidic flow chambers were incubated in a humidified incubator at 37 °C and 5% CO_2_, and the medium was exchanged every 30 min. After 2 h of static culture, the cell layer was reviewed by bright-field microscopy. As soon as the cells showed sufficient adhesion to the membrane (~ 2 to 2.5 h), the endothelialized flow chambers were connected to the model system in a sterile manner. Dynamic culture was initiated by applying a laminar flow of 0.77 mL min^−1^ to the cells with a corresponding physiological WSS of 0.5 Pa (equivalent to 5 dyn cm^−2^).^29^ The culture medium was exchanged twice weekly.

In advance of the gas transfer tests, three endothelialized membranes with ECs from different donors were each dynamically cultured for 3, 19 or 33 days. As a reference, the same number of endothelialized membranes was dynamically cultured for immunocytochemical staining.

### Venous Blood for Gas Transfer Testing

To test gas transfer rates, we used fresh, pooled and heparinized porcine blood from an abattoir. Initially, 15,000 IU L^−1^ heparin-sodium (B. Braun Melsungen), 1.8 mL L^−1^ 50% glucose solution (B. Braun Melsungen), 1.2 mL L^−1^ antibiotics solution (10 mg mL^−1^ gentamycin, Biochrom) and 6 mL L^−1^ 0.9% sodium chloride (B. Braun Melsungen) were added to the porcine whole blood.

All tests were performed with blood inlet conditions according to DIN EN ISO 7199.^6^ To meet the specifications, the heparinized blood was transferred to a simplified heart–lung machine setup comprising an open reservoir, a roller pump and an oxygenator with a heat exchanger to adjust the blood parameters according to Table 1. The hemoglobin concentration and the base excess were adjusted by adding 0.9% sodium chloride and 8.4% sodium bicarbonate (Fresenius Kabi), respectively. The oxygenator was supplied with an adjustable gas mixture of oxygen, nitrogen and carbon dioxide. Throughout the experiments, the blood parameters were checked regularly by blood gas analysis (ABL 800 Flex, Radiometer) and the settings were adjusted accordingly.

**Table 1:** Blood conditions for *in vitro* testing of oxygen and carbon dioxide transfer rates specified by DIN EN ISO 7199:2017^6^.

Parameter	Range
Hemoglobin	12 ± 1 g dL^−1^
Oxyhemoglobin	65 ± 5%
Base excess	0 ± 5 mmol L^−1^
pCO_2_	6.0 ± 0.7 kPa (equivalent to 45 ± 5 mmHg)
Temperature	37 ± 1 °C

### Gas Transfer Tests

Gas transfer tests were carried out on three endothelialized microfluidic flow chambers (*n* = 3) for each culture period (3, 19 and 33 days). Three blank RGD-PDMS flow chambers served as references. The RGD-PDMS film of the flow chamber acts as a gas exchange membrane between the microfluidic channel (blood pathway) and the gas compartment (gas pathway). For gas transfer testing, the device was perfused on the principle of counter flow (Fig. 1c).

In more detail, the blood pathway consisted of a syringe pump (LA-120, Landgraf Laborsysteme HLL) placed on a platform shaker, a 50-mL syringe (PERFUSOR, B. Braun Melsungen), extension lines (Type Heidelberger, 30 cm, Fresenius Kabi) with three-way valves for sampling (DISCOFIX, B. Braun Melsungen), the flow chamber to be tested, and a waste container. The gas pathway provided pure oxygen and was equipped with a thermal mass flow meter (TSI 4100 series, TSI) to ensure a constant gas flow rate. The blood flow rate was set by the syringe pump.

All gas transfer experiments were conducted in a climatic chamber at 37 °C. Initially, blood meeting the standards of ISO 7199 was drawn into a 50-mL syringe and connected to the blood pathway, as was the microfluidic flow chamber. Blood and gas flow were started and slowly increased to the target values of 0.77 and 500 mL min^−1^, respectively, to prevent any damage to the cell layer. Both flows were stopped immediately after sampling, which corresponds to an exposure time of approximately 20 min. The blood flow rate corresponds to a hemodynamic WSS of 2.5 Pa (assuming a blood viscosity of 3.6 mPa s).^11^ Based on our unpublished results showing sufficient stability of the cell layer after short-term blood exposure, this supraphysiological WSS was chosen in favor of higher flow rates and more accurate sampling.

With 1-mL syringes (B. Braun Melsungen), samples of at least 0.5 mL volume were taken in triplicate beginning at the outlet (arterial samples) and then at the inlet (venous samples) of the microfluidic flow chamber. Venous samples were drawn after arterial samples to avoid any impact on the blood residence time within the channel. Sampling was time-controlled to avoid accidentally affecting the flow conditions inside the microfluidic chamber. Prior to sampling, the microfluidic flow chamber was perfused with at least three times its volume to ensure an equilibrium state. Moreover, the dead volume in the sampling port was discarded. Possible air bubbles were removed and syringes were hermetically sealed. Samples were stored on ice until analysis in a blood gas analyzer, but no longer than 20 min.

### Evaluation of Gas Transfer Measurements

The blood gas analysis determines the concentration of total oxygen and total carbon dioxide in whole blood (tO_2_ and tCO_2_; the underlying calculations were previously published^3^,^25^) taking into account the respective fractions of physically dissolved and chemically bound gas. The oxygen and carbon dioxide transfer were then computed as the difference in mean gas concentrations between the inlet and outlet samples. Relating the transfer to the blood flow rate yielded the transfer rates for oxygen and carbon dioxide, as shown below:

1 $$ {\text{OTR = }}\dot{V} ( {\text{tO}}_{{ 2 , {\text{arterial}}}} - {\text{tO}}_{{ 2 , {\text{venous}}}} ) $$

2 $$ {\text{CTR = }}\;\dot{V}  ( {\text{tCO}}_{{ 2 , {\text{arterial}}}} - {\text{tCO}}_{{ 2 , {\text{venous}}}} ) $$

### Immunocytochemistry and Fluorescence Microscopy

All endothelialized RGD-PDMS samples were fixed for immunocytochemical staining with antibodies for CD31 and von Willebrand factor (vWf) as described elsewhere.^15^ Prior to immunocytochemical staining, endothelialized membranes exposed to blood were carefully rinsed with PBS containing 2% ABM to remove blood residues.

Briefly, the ECs were fixed in ice-cold methanol and nonspecific binding sites were blocked with 3% bovine serum albumin (BSA, Sigma-Aldrich) in PBS. The cells were incubated sequentially with primary antibodies against CD31 (monoclonal, mouse, P8590, Sigma-Aldrich) and vWf (monoclonal, rabbit, A0082, Dako) as well as their corresponding secondary antibodies (Alexa Fluor 594, goat anti-mouse, A11005, and Alexa Fluor 488, goat anti-rabbit, A11008, both Thermo Fisher Scientific) for 1 h each at 37 °C. Cells were counterstained with DAPI (Carl Roth). Samples were viewed by fluorescence microscopy (AxioObserver Z1; Carl Zeiss) and images were acquired using a high-resolution CCD camera (AxioCam MRm, Carl Zeiss) and AxioVision software.

### Statistical Analysis

Results are presented as means ± standard deviations (SD). Both outcome variables OTR and CTR were separately analyzed by one-way ANOVA to test for differences in the mean outcome between the four test conditions (three different culture periods and the blank reference). Data were analyzed using Excel 2016 (Microsoft Corp.) and SAS v9.4 software (SAS Institute). The statistical analysis was conducted in an explorative manner, and a *p* value below 0.05 was considered to be statistically significant (labeled *).

## Results

### Long-Term Dynamic Culture in the Model System

**Figure 2:**
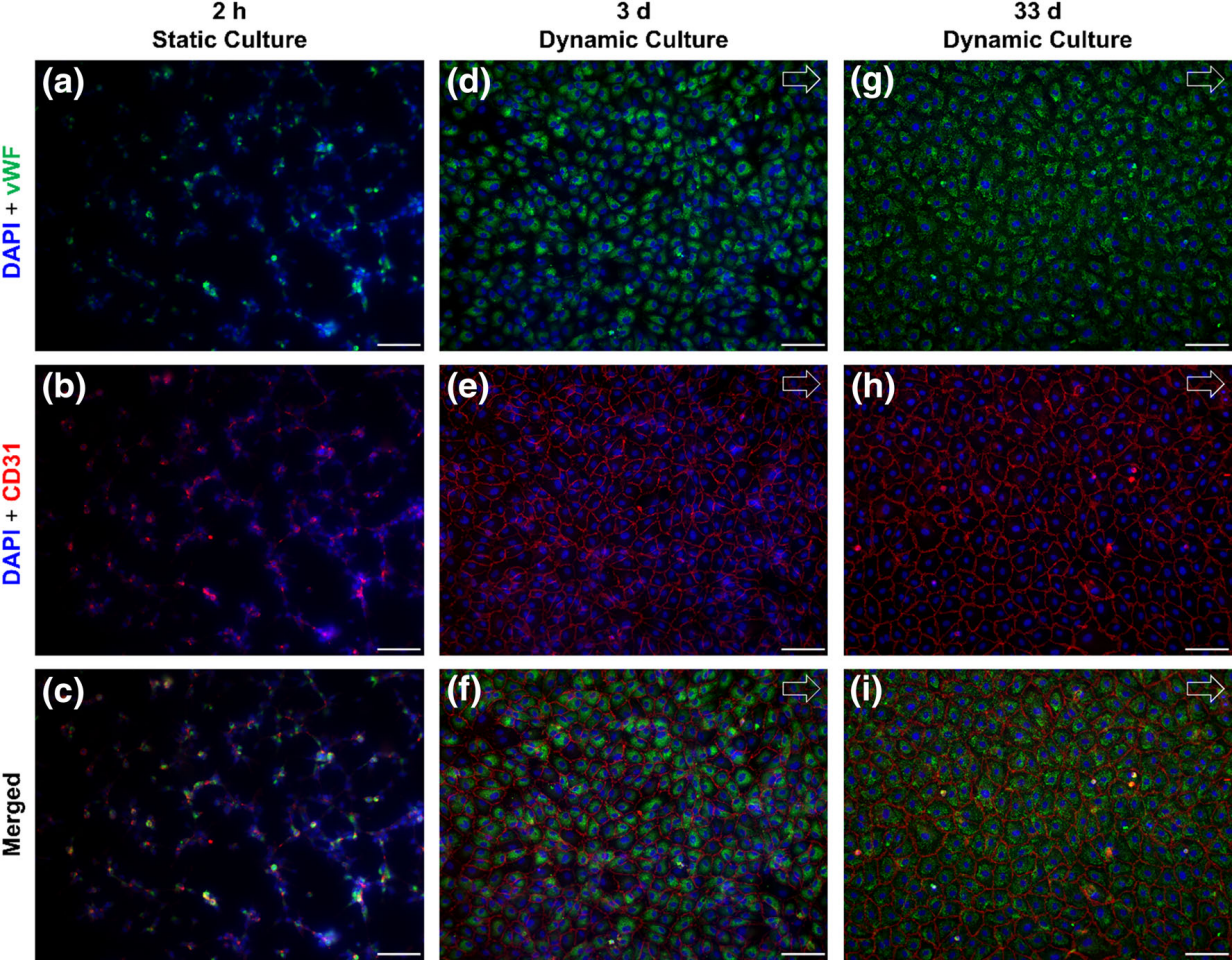
Endothelial cells seeded on RGD-PDMS after static culture for 2 h (a–c), on day 3 (d–f) and day 33 (g–i) of dynamic culture with a WSS of 0.5 Pa. Sub-confluent cell layer with minor cell aggregates and weak staining for CD31 (red) and von Willebrand factor (vWf, green) after static culture. By day 3 of dynamic culture, the cells formed a confluent cell layer and showed a more evenly-spread morphology, with no significant changes between day 3 and day 33. Cells were counterstained with DAPI (blue). Scale bars 100 *µ*m. Arrows indicate the direction of flow.

For the endothelialization of RGD-PDMS membranes, EC suspensions were transferred to the microfluidic flow chambers and cultured at 37 °C and 5% CO_2_. After 2–2.5 h of static culture, the ECs showed sufficient adhesion to the RGD-PDMS membrane to initiate dynamic culture at 0.5 Pa. Over a culture period of 33 days, the ECs maintained a confluent and integral cell layer, showing stable endothelialization under flow conditions. Figure 2 shows fluorescence microscopy images of ECs seeded on RGD-PDMS membranes after 2 h of static cultivation as well as after 3 and 33 days of dynamic culture at 0.5 Pa. Cells were stained for CD31 (red) and vWf (green), and counterstained with DAPI (blue). All samples stained positively for the two typical EC markers, confirming the endothelial phenotype. Although the cell coverage was sub-confluent and showed minor aggregation at the initiation of dynamic culture (Fig. 2c), the cells achieved confluence and displayed a more flattened morphology at a mean WSS of 0.5 Pa (Figs. 2f and 2i) after 3 days and ultimately after 33 days of dynamic culture. In addition, vWf was mainly present intracellularly in its granular form.

### Gas Transfer Testing

Gas transfer tests were carried out on three endothelialized microfluidic chambers (*n* = 3) for each culture period (3, 19 and 33 days). Three blank RGD-PDMS devices served as references. All inlet blood parameters were within the range set by ISO 7199. Moreover, there was no significant decrease in hemoglobin concentration, indicating the absence of blood sedimentation in the tubing system. The blood flow and the gas pathway allowed for uniform perfusion and did not result in lifting or bulging of the membrane.

**Figure 3:**
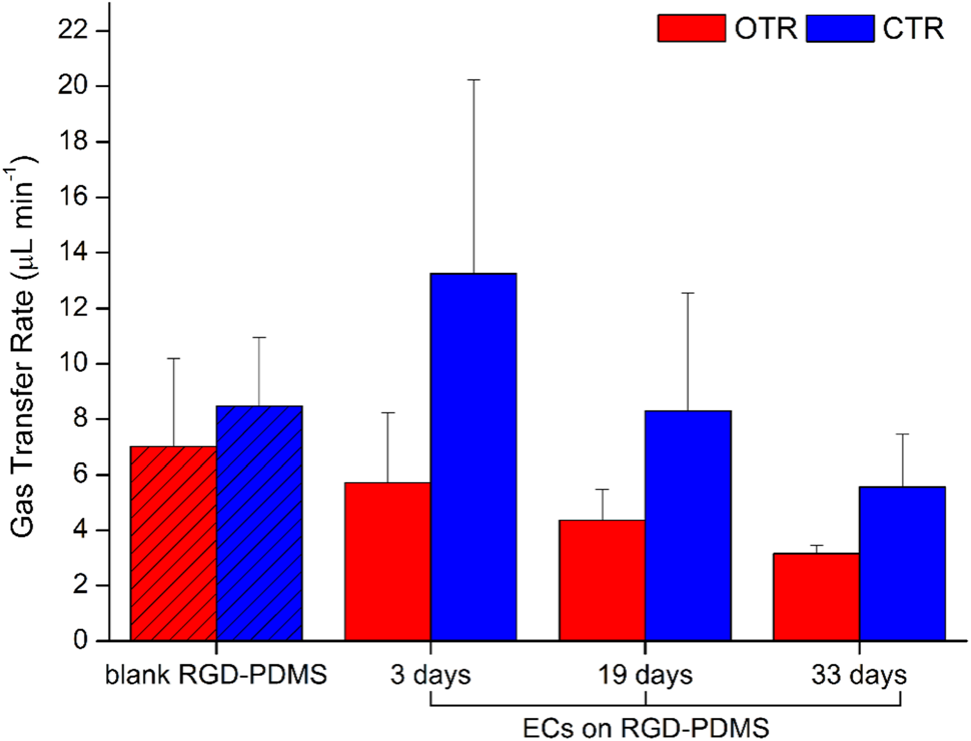
Oxygen and carbon dioxide transfer rates (OTR, CTR) of blank and cell-seeded RGD-PDMS membranes after 3, 19 and 33 days of dynamic culture at a blood flow rate of 0.77 mL min^−1^ and an oxygen flow rate of 500 mL min^−1^.

The gas transfer rates for oxygen and carbon dioxide were computed according to Eqs. (1) and (2) and are shown in Fig. 3. The three venous and arterial samples were pooled for each biological donor and each culture period, respectively, to account for variations in the sampling procedure. All samples exhibited positive OTRs and negative CTRs, corresponding to oxygenation and decarboxylation of the blood. In addition, the absolute mean OTR and CTR values decreased following cell seeding and subsequently with longer culture times, but there were no statistically significant differences between the means of OTR respectively CTR for each culture period and blank reference as determined by both one-way ANOVA’s (OTR: *F*(3,8) = 1.87, *p* = 0.2135; CTR: *F*(3,8) = 1.59, *p* = 0.2672).

**Figure 4:**
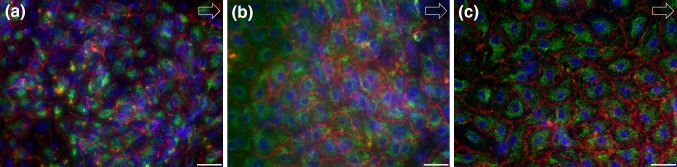
Immunocytochemical staining of endothelialized RGD-PDMS membranes for DAPI, CD31 and vWf after 3 (a), 19 (b) and 33 days (c) exposed to a maximum WSS of 2.5 Pa after blood contact. Scale bar 50 *µ*m. Arrows indicate the direction of flow.

To ensure that the gas exchange measurements were not compromised by a damaged cell layer, the samples were stained after the gas transfer tests. All endothelialized membranes from all culture periods (Fig. 4) exhibited a confluent endothelial cell layer comparable to cells cultured in standard medium (Fig. 2) after contact with blood and following exposure to a WSS of 2.5 Pa.

## Discussion

This study reports on the long-term stability and gas exchange performance of endothelialized RGD-PDMS membranes. The results have shown the promotion of endothelialization by RGD-PDMS as well as the stability of the endothelialized membranes for at least 33 days and for a maximal WSS of 2.5 Pa. Moreover, gas transfer tests following ISO 7199 provided evidence of the oxygenation and decarboxylation of the blood across endothelialized membranes with an apparent but not significant decrease of transfer rates over time.

In general, the concept of a biohybrid lung addresses the lacking hemocompatibility of current oxygenators by forming a non-thrombogenic and anti-inflammatory surface for contact with the patient’s blood. Although the endothelialization of gas exchange membranes has already been demonstrated,^9^,^15^ there have been no studies on long-term stability of endothelialized gas exchange membranes under physiological and supraphysiological flow conditions, and little is therefore known about their gas exchange performance in blood.

This study was designed to close the knowledge gap by investigating the long-term stability of an endothelialized RGD-PDMS membrane both under well-defined physiological WSS conditions and under significantly elevated WSS in a gas transfer test performed with porcine blood and in accordance with ISO 7199. Although highly-porous PMP and polypropylene (PP) hollow fiber membranes are widely used in ECMO applications, we investigated RGD-PDMS membranes for the use in a biohybrid lung. In a first evaluation of this membrane system, a flat RGD-PDMS membrane, in contrast to a hollow fiber membrane, was used to facilitate monitoring of the endothelial cell layer as well as the application of a laminar flow with a well-defined wall shear stress. In the context of a biohybrid lung, PDMS is particularly suitable as a plasma-tight ultra-thin top layer for a composite gas exchange membrane with a macroporous supporting structure, as has been shown by the development of intravascular oxygenator (IVOX) devices with silicone coatings as thin as 1 *µ*m,^18^ or even 0.2 *µ*m.^34^ For the design of a biohybrid lung, PDMS has excellent gas permeability and hemocompatibility and is amenable to various surface modifications.^1^,^2^,^12^,^16^,^17^,^36^ We found that ECs in dynamic culture were proliferative and reached confluence on PDMS membranes within 3 days, as confirmed by immunocytochemistry (Fig. 2f). This was achieved despite the short initial seeding process involving only 2 h of static culture and a sudden transition to the final flow rate. In previous studies, ECs were prepared by allowing longer static culture periods^37^ or by applying flow preconditioning^27^ to ensure the development of a confluent and flow-stable cell layer. Considering the high packing density of gas exchange membranes in a biohybrid lung device, which limits nutrient supply during static culture, the flow stability of the EC layer after only 2 h in static culture indicates the particular suitability of RGD-conjugated PDMS membranes in terms of the initial cell adhesion process.

Furthermore, our results not only show the overall durability and suitability of the surface-modified PDMS membranes for chronic lung support, but also demonstrate the long-term stability of the EC layer on top of the membrane, even under flow conditions. In addition, the presence of intracellularly stored vWf in its granular form indicates on the non-thrombogenic status of the endothelial cells over the entire culture period.^7^,^19^ A largely intact EC layer on heparin/albumin-coated PMP membranes was previously reported after a maximum of 14 days under undefined flow conditions.^20^ In contrast, our investigation successfully tested a culture period of more than 4 weeks under well-defined flow conditions. In detail, the endothelialized RGD-PDMS membranes were cultured with a WSS of 0.5 Pa for a maximum of 33 days and during gas exchange performance testing with a WSS of 2.5 Pa. Although the intended use of a biohybrid lung ranges from several months to even years, a culture period of 33 days was chosen for our preliminary study to exceed the clinically relevant 4-week approval of current oxygenators. In addition, the first flow condition with a WSS of 0.5 Pa was selected to approximate the physiological WSS of 0.5 Pa.^29^ This value is also similar to the mean WSS of 0.7 Pa seen in hollow-fiber membrane oxygenators.^33^ Nevertheless, higher peaks of WSS may occur in current oxygenators and their influence on endothelialization must be addressed in future studies. However, the short-term exposure to a supraphysiological WSS of 2.5 Pa during gas transfer testing did not affect the integrity of the EC layer. This is also consistent with the observation of flow preconditioning and its positive influence on cell retention.^27^ Moreover, the gas pathway was not constantly flushed with pure oxygen but only during gas transfer testing. The exposure to pure oxygen causes oxidative stress in endothelial cells and lead to a decrease in cell density.^23^ Therefore, pure oxygen cannot easily be used as a permanent sweep gas. However, this is not necessarily required for all applications of a biohybrid lung. The extracorporeal carbon dioxide removal (ECCO_2_R) in hypercapnic patients (such as COPD patients) may use room air as sweep gas, which would reduce the oxidative stress placed on the endothelialization and in turn also lowers the risk of delamination or activation of the endothelial cells. Therefore, we do not consider this a limitation to this study. Furthermore, it has to be noted that common to other studies cell culture medium was used for long-term dynamic culture as an alternative to blood and that endothelial cell behavior may differ for prolonged exposure to blood. Together with the small sample size of three biological replicates, this represents a limitation of this preliminary study and needs to be addressed in further research.

The investigation of endothelialized membranes to determine flow stability and gas exchange performance must accommodate the special requirements for *in* *vitro* blood testing as well as the resulting limitations. Accordingly, we considered the non-linear gas binding characteristics of whole blood and the various influencing factors^31^ were taken into account. With the help of a simplified heart–lung machine setup, the blood inlet conditions were kept within a narrow measuring range (Table 1) for the entire duration of the test, but at the expense of a higher blood volume demand. Given that the investigation of flow stability and gas exchange performance does not necessitate the use of human blood, porcine blood was chosen for better availability. Pure oxygen was used as a sweep gas to increase the partial pressure difference over the endothelialized membrane, thus achieving sufficient gas exchange despite the small gas exchange surface area of the microfluidic flow chamber. The undamaged and integral endothelial cell layer after gas transfer testing confirmed the validity of this robust experimental approach with short-term exposure to porcine blood (~ 20 min) and pure oxygen despite the oxidative stress and the use of xenogeneic blood. Therefore, use of porcine blood was considered a justifiable limitation.

Gas transfer testing demonstrated the oxygenation and decarboxylation of whole blood over the blank as well as the endothelialized membrane during up to 33 days of dynamic culture. Although the absolute gas transfer rates are in the range of several *µ*L min^−1^, a normalization to 1 m^2^ of gas exchange surface facilitates a theoretical comparison to commercial oxygenators. For a 100 *µ*m non-porous RGD-PDMS membrane, this yields an OTR_normalized_ of 28 mL min^−1^ m^−2^. In contrast, highly-porous PMP hollow fiber membrane oxygenators can provide OTR_normalized_ of more than 200 mL min^−1^ m^−2^, with 215 mL min^−1^ m^−2^ for the 1.9 m^2^ Hilite 7000 LT (Medos) and 236 mL min^−1^ m^−2^ for the 1.8 m^2^ Quadrox D (Maquet).^4^ Although the OTR_normalized_ differ, the results have to be put into perspective. As previously discussed, the prospective design of a biohybrid lung, unlike our current microfluidic model system, would foresee the use of a composite membrane consisting of a 1-*µ*m PDMS membrane and a macroporous and highly gas permeable supporting structure. While the supporting structure would provide mechanical stability, the gas exchange performance of the composite membrane would be primarily defined by the PDMS. With a PDMS membrane 100 times thinner than the current membrane and a suitable supporting structure, the OTR_normalized_ could theoretically yield values in the range of 280 mL min^−1^, which would exceed the OTR_normalized_ of commercially available oxygenators.

For the gas transfer rates of endothelialized membranes on day 3, our results agree with previous studies, which showed that the cell layer caused little if any decline in gas transfer rates.^5^,^9^,^13^,^24^ Furthermore, a small, albeit not significant, decrease in transfer rates over longer culture periods (19 and 33 days) is apparent. Within the scope of this study, the microfluidic flow chamber facilitated the investigation of the cell-membrane interface under controlled and well-defined experimental conditions. These measures to increase reproducibility, however, are to the detriment of gas exchange properties. Gas exchange is a key performance indicator of a biohybrid lung and it is not yet clear, whether the gas transfer decrease over time may be an inherent property of the maturing endothelium or is caused by other effects. Hence, a comprehensive evaluation of gas exchange performance needs to be addressed in further studies with an adapted laboratory prototype of a biohybrid lung.

Such a biohybrid lung prototype should be equipped with an appropriate surface area of a macroporous composite membrane with a plasma-tight thin PDMS coating that will achieve sufficient gas transfer rates, for example in the context of an animal experiment to circumvent the limitations of *in vitro* blood trials. Furthermore, such a device would also be highly interesting for hemocompatibility studies to investigate the interdependent and complex aspects of thrombogenicity and inflammation that have not been investigated in the scope of this study. Nevertheless, this study demonstrates that the endothelialized RGD-PDMS membrane system tolerates the stresses and strains placed on the endothelial layer during ISO 7199-compliant gas transfer testing, thus showing the suitability of our approach for use in a biohybrid lung for three independent biological donors.

This study investigated the long-term stability and gas exchange performance of endothelialized RGD-conjugated PDMS membranes in a model system for chronic lung support in a biohybrid lung. The results affirmed the overall durability and suitability of the membrane system and demonstrated the long-term stability of the endothelial layer for at least 33 days at a physiological WSS of 0.5 Pa. The study parameters were chosen according to the physiological WSS of HUVECs, which is similar to the mean square WSS of hollow-fiber membrane oxygenators and approximates the maximum approval period of today’s oxygenators (30 days). Short-term exposure to a significantly higher WSS of 2.5 Pa during gas exchange tests showed no effect on the integrity of the EC layer. Despite the limitations of a microfluidic model system, a small sample size and *in vitro* blood trials, the gas transfer tests according to ISO 7199 provided evidence for the oxygenation and decarboxylation across endothelialized membranes with a decrease of transfer rates for longer culture periods (19 and 33 days). Although the gas exchange characteristics need to be examined in more detail and further investigations are necessary, our results demonstrate the general suitability of RGD-PDMS membranes for biohybrid lung applications, which might enable long-term support of patients with chronic lung failure in the future.

## Abbreviations

ECs: Endothelial cells
RGD-PDMS: RGD-conjugated polydimethylsiloxane
WSS: Wall shear stress
vWf: von Willebrand factor
PBS: Phosphate-buffered saline
OTR: Oxygen transfer rate
CTR: Carbon dioxide transfer rate
